# Metabolomic biosignature differentiates melancholic depressive patients from healthy controls

**DOI:** 10.1186/s12864-016-2953-2

**Published:** 2016-08-23

**Authors:** Yashu Liu, Lynn Yieh, Tao Yang, Wilhelmus Drinkenburg, Pieter Peeters, Thomas Steckler, Vaibhav A. Narayan, Gayle Wittenberg, Jieping Ye

**Affiliations:** 1 Department of Computer Science and Engineering, Center for Evolutionary Medicine and Informatics, The Biodesign Institute, Arizona State University, Tempe, AZ 85287 USA; 2 Janssen Research & Development, LLC, 3210 Merryfield Row, San Diego, CA 92121 USA; 3 Janssen Research & Development, LLC, Turnhoutseweg 30, 2340 Beerse, Belgium; 4 Janssen Research & Development, LLC, 1125 Trenton-Harbourton Road, Titusville, NJ USA

**Keywords:** Major depressive disorder, Melancholic depression, Metabolomics, Classification, Biomarker

## Abstract

**Background:**

Major depressive disorder (MDD) is a heterogeneous disease at the level of clinical symptoms, and this heterogeneity is likely reflected at the level of biology. Two clinical subtypes within MDD that have garnered interest are “melancholic depression” and “anxious depression”. Metabolomics enables us to characterize hundreds of small molecules that comprise the metabolome, and recent work suggests the blood metabolome may be able to inform treatment decisions for MDD, however work is at an early stage. Here we examine a metabolomics data set to (1) test whether clinically homogenous MDD subtypes are also more biologically homogeneous, and hence more predictiable, (2) devise a robust machine learning framework that preserves biological meaning, and (3) describe the metabolomic biosignature for melancholic depression.

**Results:**

With the proposed computational system we achieves around 80 % classification accuracy, sensitivity and specificity for melancholic depression, but only ~72 % for anxious depression or MDD, suggesting the blood metabolome contains more information about melancholic depression.. We develop an ensemble feature selection framework (EFSF) in which features are first clustered, and learning then takes place on the cluster centroids, retaining information about correlated features during the feature selection process rather than discarding them as most machine learning methods will do. Analysis of the most discriminative feature clusters revealed differences in metabolic classes such as amino acids and lipids as well as pathways studied extensively in MDD such as the activation of cortisol in chronic stress.

**Conclusions:**

We find the greater clinical homogeneity does indeed lead to better prediction based on biological measurements in the case of melancholic depression. Melancholic depression is shown to be associated with changes in amino acids, catecholamines, lipids, stress hormones, and immune-related metabolites. The proposed computational framework can be adapted to analyze data from many other biomedical applications where the data has similar characteristics.

**Electronic supplementary material:**

The online version of this article (doi:10.1186/s12864-016-2953-2) contains supplementary material, which is available to authorized users.

## Background

Major Depressive Disorder is the most common mental illness, affecting an estimated 350 million people worldwide [1]. For many, MDD is a lifelong illness consisting of recurrent episode, each of which may cause disability and severely interfere with an individual’s everyday life, and greatly increase the risk of suicidal behavior [2]. Current antidepressants are fully effective in only about a third of patients, with another third partially responding. Thus there is a tremendous need to identify novel therapies to help those not served well by today’s treatment strategies. This can only come with a deeper understanding of the biology and the biological heterogeneity of MDD.

The identification of replicable biomarkers differentiating patients with MDD from healthy controls has lagged behind other diseases. This is reflected in a mega-analysis of GWAS data performed by the Major Depressive Disorder Working Group of the Psychiatric GWAS Consortium on 9240 MDD cases and 9519 controls which identified no replicable markers, despite the detection of significant markers in 94 % of other disease tested in populations of the same size [3]. A key factor likely hindering the discovery of biomarkers for or prediction of MDD compared with other diseases is high degree of clinical and biological heterogeneity [4–11]. In the DSM V diagnostic manual, MDD is defined by patients having at least one of the two core depressive symptoms, ‘depressed mood’ and ‘anhedonia’, and at least 5 of 9 overall symptoms. This definition alone leads to heterogeneity based on the combination of symptoms endorsed by any given patient. Even within individual symptom items there is heterogeneity with criteria such as “gaining weight or losing weight”, “hypersomnia or insomnia”, “psychomotor agitation or retardation”. It would be surprising if such clinical heterogeneity were not also reflected in the biology of MDD.

Two patient groups easily separable based on clinical features are those with melancholic depression and those with anxious depression. Melancholic depression is characterized by pervasive anhedonia, lack of reactivity to circumstances, depressed mood of a distinct quality, and typical vegetative symptoms such as appetite or weight loss, early morning awakening, worse mood in the morning [4, 12, 13]. The prevalence of melancholic depression among depression population is around 25-30 % [14]. Melancholic depression is found to exhibit stronger response to physical treatments but weaker response to psychotherapy or placebos compared to other subtypes of depression [15–17]. Anxious depression is characterized by the co-occurrence of MDD with anxious symptoms, possibly subthreshold for diagnosis of an anxiety disorder. Anxiety during an MDD episode has been widely studied as a predictor of a more severe course of illness, and poorer patient outcomes [18–20].

Here we report results on the Janssen-BRC metabolomics data set, consisting of 97 healthy control and 90 MDD subjects, of which 21 suffer melancholic depression and 58 from anxious depression. In this work, our goals are three-fold. First, we test the hypothesis that more clinically homogeneous groups of MDD patients are easier to predict from healthy controls than the entire MDD group using blood metabolomics data. Second, we develop a novel method for building maximally predictive and robust machine-learning classifiers that retain information on the correlation structure of the metabolomics data to ease biological interpretation. Third, we use this framework to describe the metabolomics biosignature of melancholic depression.

## Methods

### Data description

The Janssen-BRC metabolomic data set was part of a case-control study of MDD designed to detect biomarkers of depression and subtypes of depression by investigating various data sources, such as patients’ demographic information, psychophysiological and neuropsychological indices, and molecular profiling. Volunteers were recruited nationwide by Brain Resource Company (BRC) in Australia. Patients were designated as MDD if they scored > = 14 on the Hamilton Depression Rating Scale-17 (HAMD17). Within MDD subjects, patients were designated as melancholic depressed if they additionally scored > =8 on the CORE scale [21–24]. The CORE scale for melancholic depression consists of a 18-item scale with each item rated on a 4-point scale (0-3) by clinicians [25, 26]. Patients were designated as anxious depressed the number of comorbid anxiety disorders on the M.I.N.I. International Neuropsychiatric Interview > 0. Based on these diagnostic criteria, the data set consists of 97 healthy control and 90 MDD subjects, of which 21 suffer melancholic depression and 58 from anxious depression. One healthy control subject was not evaluated for anxiety, was therefore not included in the analysis of healthy control vs. anxious depressed out of caution. For detailed statistics of the samples used in our study, readers may refer to Table 1.

**Table 1:** Sample statistics of metabolite data for the classification of melancholic depression. Mean statistics are reported with standard deviation, minimum value, and maximum value in the parenthesis

	HC	MDD	Melancholic depressed	Anxious depressed
# of samples	97	90	21	58
Age	38.49 (14.72, 16.25-74.38)	39.70 (14.10,18.66-76.75)	40.59 (12.69, 19.86-68.97)	39.78 (13.95, 18.66-68.97)
Gender(%female)	60.82 %	63.33 %	57.14 %	60.34 %
Education	14.85 (2.41, 7-18)	13.87 (2.95, 3-18)	13.81 (3.72, 3-18)	14.36 (2.55, 9-18)
HAMD	0.28 (0.72, 0-4)	21.90 (3.49, 18-34)	24.57 (4.46,19-34)	22.14 (3.61,18-34)
CORE	-	5.43 (4.21, 0-23)	11.24 (3.96, 8-23)	5.71 (4.50, 0-23)

This study was approved by Institutional Review Board (IRB)/Independent Ethics Committee (IEC) including the protocol and written informed consent form at each trial site. Written consent was obtained from participants prior to enrollment in the study.

### Metabolomics sample

A 20 ml sample of plasma was collected into EDTA-containing tubes from 99 depressed subjects and 100 healthy controls (gender and age matched) collected from four recruitment sites at BRC, Australia. Samples were stored at BRC at -20 °C prior to shipment after which they were shipped on dry ice and stored at -80 °C. Plasma samples were profiled by Metanomics Health GmbH. Plasma samples were extracted by a proprietary method and separated into four fractions (lipid and polar fractions) prepared for Gas chromatography–mass spectrometry (GC-MS) and Liquid chromatography–mass spectrometry (LC-MS/MS). For GC-MS analytics the samples were sequentially derivatized before measurement. In LC-MS/MS analysis a metanomics proprietary technology was applied which allows target and high sensitivity MRM (Multiple Reaction Monitoring) profiling in parallel to full screen analyses.

Data were acquired on 272 peaks: 160 peaks mapped to known metabolites and the remaining 112 unknown. Data were centered to the median of healthy control samples, and log-transformed to assure normal distribution of the data.

### Correction of storage time effects

The concentration of many metabolites is known to change as a function of storage time. We removed 44 metabolites reported by Metanomics Health as having sensitivity to storage time in the same direction as the change observed in our data set, leaving us with 228 metabolites. Those not deemed sensitive to storage may still have residual storage time effects. To assess this, we calculate the *p*-values of bivariate correlations between metabolites and the storage time, and assess their deviation from the null distribution (that expected under the null hypothesis of no metabolite associated with the storage time) in a Quantile-Quantile (QQ) plot (Additional file 1: Figure S1 (left)). The calculated *p*-values (-log transformed) for metabolites are sorted in descending order and plotted against the values sampled from the expected uniform distribution of *p*-values. The strong deviations from the straight line suggest that either the null distribution is incorrect or that there exists a true association. In our study, we observe that the linear relationship with the storage time at -20° continues up to 200 days, while the effects of the storage time are skewed after day 200. We thus remove samples stored for more than or equal to 200 days.

To control for this effect, we correct the metabolite data by taking the residuals of linear regression models on storage time at -20° [27]. We build the regression model on healthy controls only, and then apply the model to all subjects in order to avoid the removal of some disease-related effects as suggesied in [28].

Specifically, let *X* be one column of the metabolite feature vector (one metabolite) to be corrected, and *T* be the column vector of the storage time at -20°. The lengths of X and T equal the sample size. Let *X*_*H*_ and *T*_*H*_ denote the feature and storage time vectors of healthy controls respectively. Then, we correct the metabolite features as follows.

1. We build the following linear regression model on the 97 healthy control samples

$$ {X}_H\kern0.5em =\kern0.5em {\beta}_0\kern0.5em +\kern0.5em {T}_H{\beta}_1, $$

where *β*_1_ and *β*_0_ stand for the effect of storage time *T* on *X*, and the bias, respectively. We can estimate the parameters as follows:

$$ \left(\begin{array}{c}\hfill {\beta}_0\hfill \\ {}\hfill {\beta}_1\hfill \end{array}\right)\kern0.5em =\kern0.5em {\left({S}^TS\right)}^{-1}{S}^TX, $$

where *S* is a two-column matrix with the first column being a vector of ones and the second column being *T*. Once *β*_1_ is determined, the corrected metabolite feature *X*_*c*_ will be

$$ {X}_c\kern0.5em =\kern0.5em X\kern0.5em -\kern0.5em T{\beta}_{1\cdot } $$

2) We apply the above storage time correction on all subjects, and repeat this procedure for all metabolite features.

After correction, the strong deviation is removed from the QQ plot (Additional file 1: Figure S1 (right)) of the *p*-values of bivariate correlation between metabolite features and storage time, which indicates the effectiveness of the correction approach.

### Imputation of missing values

Among the 187 samples used in our study, 1.10 % of the feature values are missing. We impute the missing values by several different approaches:

- *halfMin:* Impute the missing values by half of the minimum value in the corresponding feature. The assumption behind this method is that most of the missing values are too small to be detected, and therefore a simple approach is to replace the missing entries with reasonably small values. For methods such as GC/MS and LC/MS where nonlinear maps must be aligned to match peaks across samples, it may be a poor assumption that a missing value corresponds to a value below the limit of quantification, because in some instances a missing value may be the result of a misaligned, though possibly large, peak which does not get counted.
- *kNN3:* Impute the missing values by the k-nearest neighbor method (kNN). kNN imputes a missing value with a weighted average of the top k nearest-neighbor columns (*k* = 3 was used here). The weights used in kNN are inversely proportional to the distances from the neighbor columns.
- *EM:* Impute the missing values by the expectation-maximization (EM) method [29]. Under the assumption that the data matrix is Gaussian distributed, EM algorithm imputes the missing values with conditional expectation values by iteratively estimating the mean and covariance matrix from incomplete data and maximizing the likelihood of the available data.
- *SVD:* Impute the missing values by the Singular Value Decomposition (SVD) method. The SVD method, assuming the data matrix is low-rank, imputes the missing values by iteratively updating the data matrix with low-rank approximations.

In our study, all the input data matrices are normalized with zero mean and unit standard deviation before feature selection or classification. The distributions of original and imputed values of four metabolite features (Glyoxylate ratio, Caffeine ratio, Elaidicacid ratio and Indole 3 propionic acid ratio) are shown in Additional file 1: Figure S2. The distribution of the values imputed by kNN3, EM and SVD are very similar to that of original data while the halfMin method yields an imputed data with more small values as it assumes that the missing values are too weak to be observed. For our primary results reported we use kNN3 and contrast with halfMin to compare the effect on classifier performance.

### Cluster representation

Recent studies on statistical learning show that advanced feature learning algorithms like Lasso may fail to select important but highly correlated features simultaneously, and show that the clustered Lasso may lead to improved prediction and feature selection [30]. In clustered Lasso, we first apply clustering on the features to identify a set of feature groups. Then, we construct a reduced dataset consisting of cluster-representatives, which refer to the averages of the features from the same cluster. In our experiments, we first cluster the features into 100 groups by two clustering algorithms, K-means and hierarchical clustering, and then generate a 100-dimensional dataset with each feature being the centroid of each cluster. The distance used in K-means was Euclidean distance while the inter-cluster distance used in hierarchical clustering was the maximum correlation between points in two different clusters. For each cluster, the cluster centroid is calculated, and this is the new feature which is then used for classification.

We show the QQ plots of the *p*-values in the two-sample *t*-test are improved through feature clustering (Additional file 1: Figure S3). Although all the *p*-values obtained on raw features seem to be non-significant except for the minimum one, there exist strong signals for the clustering representatives (including K-means and Hierarchical Clustering). This is because QQ plot assumes that the *p*-values are independent to each other while the raw metabolite features are correlated to each other and the clustering on features can effectively mitigate the correlation.

### An ensemble feature selection framework (EFSF)

Figure 2c illustrates the feature selection + ensemble framework used in our study and we name the process the Ensemble Feature Selection Framework (EFSF)

Ensemble learning is a general and powerful framework in machine learning. The underlying philosophy of ensemble learning is to build a learner by combining a collection of base learners [31]. Ensemble learning can be divided into two tasks: 1) building a set of base learners from the training data; 2) combining the base learners to produce the predictor. The majority voting scheme in our study is one type of the ensemble learning methods. The base learners (e.g., SVM, Random Forest) are first trained from each undersample, and their predictions on testing data are then combined to form the final prediction which decides the output that has the majority (i.e., more than half the votes).

#### Undersampling

In our study, the ratio between the number of melancholic depressive patients and the number of the healthy controls is around 1:5, which is shown in Table 1. Traditional machine learning methods are less effective for such severely imbalanced data set as the classifier trained in such case will be biased towards the majority class. We adopt a very effective and commonly used approach, called “undersampling”, to deal with the imbalance problem [32–34]. In our case, we have a relatively large number of negative samples (e.g., healthy controls) and a relatively small number of positive samples (e.g., melancholic depressive patients). During the training stage, we randomly select a subset of negative samples with size equal to the total number of positive samples, and build a classifier on the combination of the positive samples and the undersampled negative samples. For instance, if there are 90 negative samples and 16 positive samples available in the training set, then 16 out of 90 negative samples will be randomly selected, which is combined with the 16 positive samples to form a training set to build a classifier. To reduce the variability of random undersampling and further enhance the performance, we repeat the undersampling procedure 30 times in the experiment and thus build 30 classifiers during the training process. Finally, we combine all the *k* classifiers via majority vote as the final prediction.

#### Feature selection

To identify the most predictive features, we apply feature selection. Since we adopt the undersampling strategy in the training process, we do feature selection on each undersampled data set and combine feature selection results to generate a final ranking of features. We employ both univariate and multivariate feature selection methods in this study including *T*-test [35], Fisher’s Score [36], Gini Index [37] and Stability Selection [38].

#### Classification

Two different widely used classifiers were tested in this study: Support vector machines (SVM) and Random Forest (RF). SVMs were first introduced in 1992 [39]. In the task of binary classification, SVM separates the two classes of data points by determining a boundary with a hyperplane which maximizes the margin of the boundary. The margin is defined as the width that a boundary could be increased by before hitting a data point. SVMs can easily build non-linear classifiers by adopting the kernel tricks [39] to implicitly map the input data into high-dimensional non-linear feature space where the data are more easily separable. In RF, the learning approach models a predictor that averages a collection of de-correlated decision / regression trees [31, 40]. In random forest, the algorithm makes a large set of bootstrap samples and builds decision / regression trees on them by selecting the best split point among a random subset of features. The final model of the random forest is the ensemble (average) of the trees grown in the bootstrap samples. Random forest takes advantage of the bootstrap aggregation to effectively reduce the high-variance made by the trees and shown success in a wide range of applications [31, 40].

In our experiments, we report the classification performances obtained from 10-fold cross validation. We randomly divide the data into 10 sets of equal size, and, holding out one set for testing, use the remaining 9 sets for training. The EFSF is applied on the training data and the learned models are used for prediction. Each set is used for testing once and thus the training and testing procedure is repeated 10 times.

### Classification performance measures

In our experiment, we treat melancholic depressed patients as positive samples and healthy controls as negative samples. Because the dataset is highly imbalanced, the commonly used performance measure, i.e., classification accuracy, is not sufficient. In addition to accuracy, we also reported both sensitivity and specificity which measure the proportions of positive and negative samples classified correctly. Specifically,

$$ \mathrm{sensitivity}\kern0.5em =\kern0.5em \frac{\mathrm{number}\kern0.5em \mathrm{of}\kern0.5em \mathrm{true}\kern0.5em \mathrm{positives}}{\mathrm{number}\kern0.5em \mathrm{of}\kern0.5em \mathrm{true}\kern0.5em \mathrm{positives}\kern0.5em +\kern0.5em \mathrm{number}\kern0.5em \mathrm{of}\kern0.5em \mathrm{false}\kern0.5em \mathrm{positives}} $$

and

$$ \mathrm{specificity}\kern0.5em =\kern0.5em \frac{\mathrm{number}\kern0.5em \mathrm{of}\kern0.5em \mathrm{true}\kern0.5em \mathrm{negatives}}{\mathrm{number}\kern0.5em \mathrm{of}\kern0.5em \mathrm{true}\kern0.5em \mathrm{negatives}\kern0.5em +\kern0.5em \mathrm{number}\kern0.5em \mathrm{of}\kern0.5em \mathrm{false}\kern0.5em \mathrm{negatives}}. $$

In our experiment, the classification performance measures (i.e., accuracy, sensitivity and specificity) are obtained from 10-fold cross-validation, described above.

### Permutation testing

In order to demonstrate the strength of the signals discovered in our study, we propose to use the framework of permutation testing [41, 42] to validate the learning results. In the permutation test [41, 42], sample labels (i.e. Melancholic Depressed, Healthy Control) are randomly permuted and the trained classifiers are tested on the permuted data set. The permutation-based *p*-value is defined as the fraction of randomized samples where the classifier performs better in the original data than in the permuted data, and is computed as:

$$ p\kern0.5em =\kern0.5em \frac{\left|\left\{D\hbox{'}\kern0.5em \in \widehat{D}:e\left(f,\kern0.5em D\hbox{'}\right)\kern0.5em \le \kern0.5em e\left(f,\kern0.5em D\right)\right\}\right|\kern0.5em +\kern0.5em 1}{K\kern0.5em +\kern0.5em 1}, $$

where $\widehat{D}$ is a set of *K* randomized (permuted) data sets *D* ' of the original data *D*, *f* is the trained classifier, and e represents the error function.

### Network visualization

Metabolites from the top 15 ranked clusters in the K-means clustering were analyzed using IPA (Ingenuity® Systems, www.ingenuity.com) for visualization and metabolite annotation. Metabolites were mapped to KEGG identifiers using the Human Metabolome Database (version 3.5, http://www.hmdb.ca/); KEGG ID’s were used as input in IPA. In some cases, the identity of metabolites was too specific for mapping to the KEGG database. Where possible, metabolites were assigned a KEGG ID, which encompasses a class of molecules, e.g. triacylglycerides. A union of 76 metabolites from the 4 feature selection methods was used as input. Of the 76 metabolites, 56 were mapped to KEGG.

## Results and discussion

### Classification performance on MDD subtypes

**Fig. 1:**
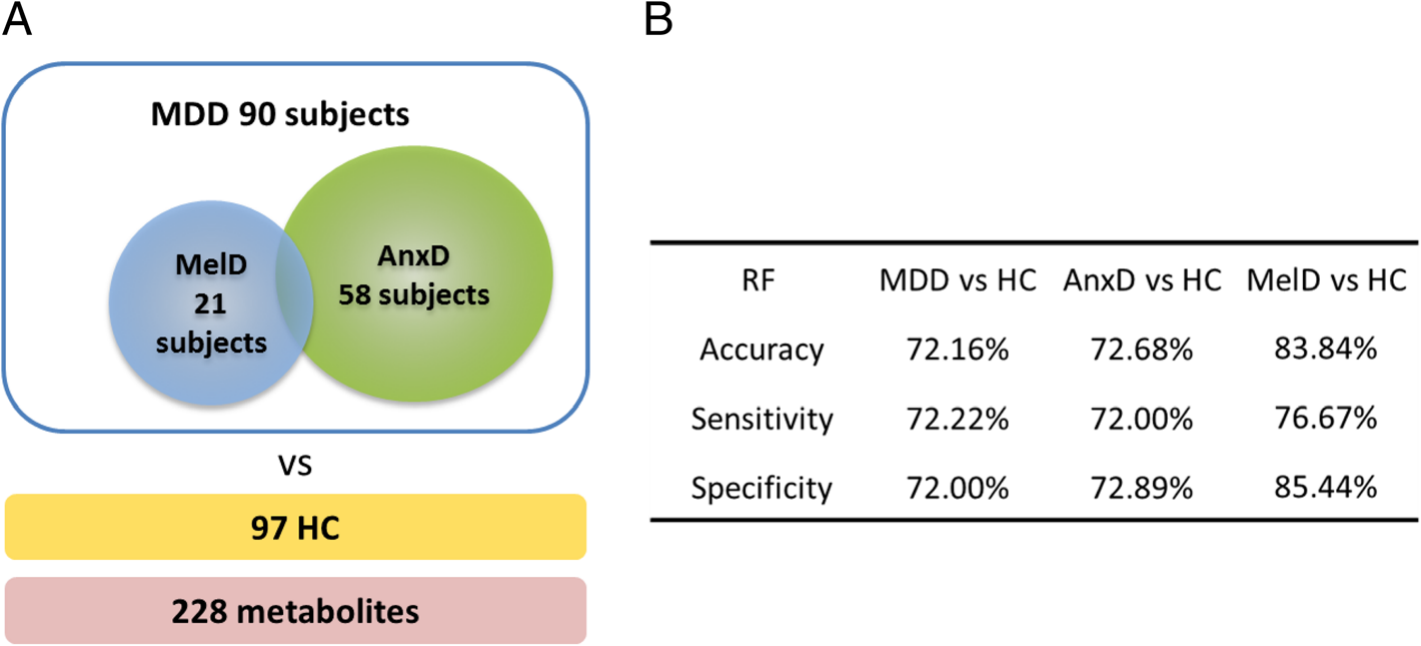
Metabolites classify Melancholic Depression from Healthy Controls with greater accuracy MDD as a whole or Anxious Depression. **a** Classifiers for 90 MDD, 58 Anxious Depressed, and 21 Melancholic Depressed subjects were trained against 97 HC subjects (96 for the Anxious Depression classification, as described in the Methods). **b** The table includes results using kNN imputation, Random Forest classification using individual metabolites as features, and the feature selection method which resulted in highest accuracy (Fisher, Gini, *T*-test or Stability) for each comparison

We first compared the performance of the Random Forest classifier using kNN3 imputation using individual metabolites (228 features) on three different classification tasks illustrated in Fig. 1.: (1) MDD vs. Healthy Control, (2) Anxious Depressed vs. Healthy Control, (3) Melancholic Depressed vs. Healthy Control. The classifier performance was higher for Melancholic Depressed patients than for the other two subgroups (Fig. 1b), suggesting that Melancholic Depressed patients may be more homogenous at the biological level (at least the blood metabolome) and easer to predict using a metabolomics biosignature. That Anxious Depressed subjects were not predicted with as high a degree of accuracy could mean that either there is less of a signal in the blood metabolome associated with symptoms of anxious depression, or alternatively that within that designation, there is still a considerable degree of biological heterogeneity. For the remainder of this work we focus on building a robust classifier for melancholic depression, and to describe the biology underlying the biosignature.

### A robust classifier for melancholic depression

**Fig. 2:**
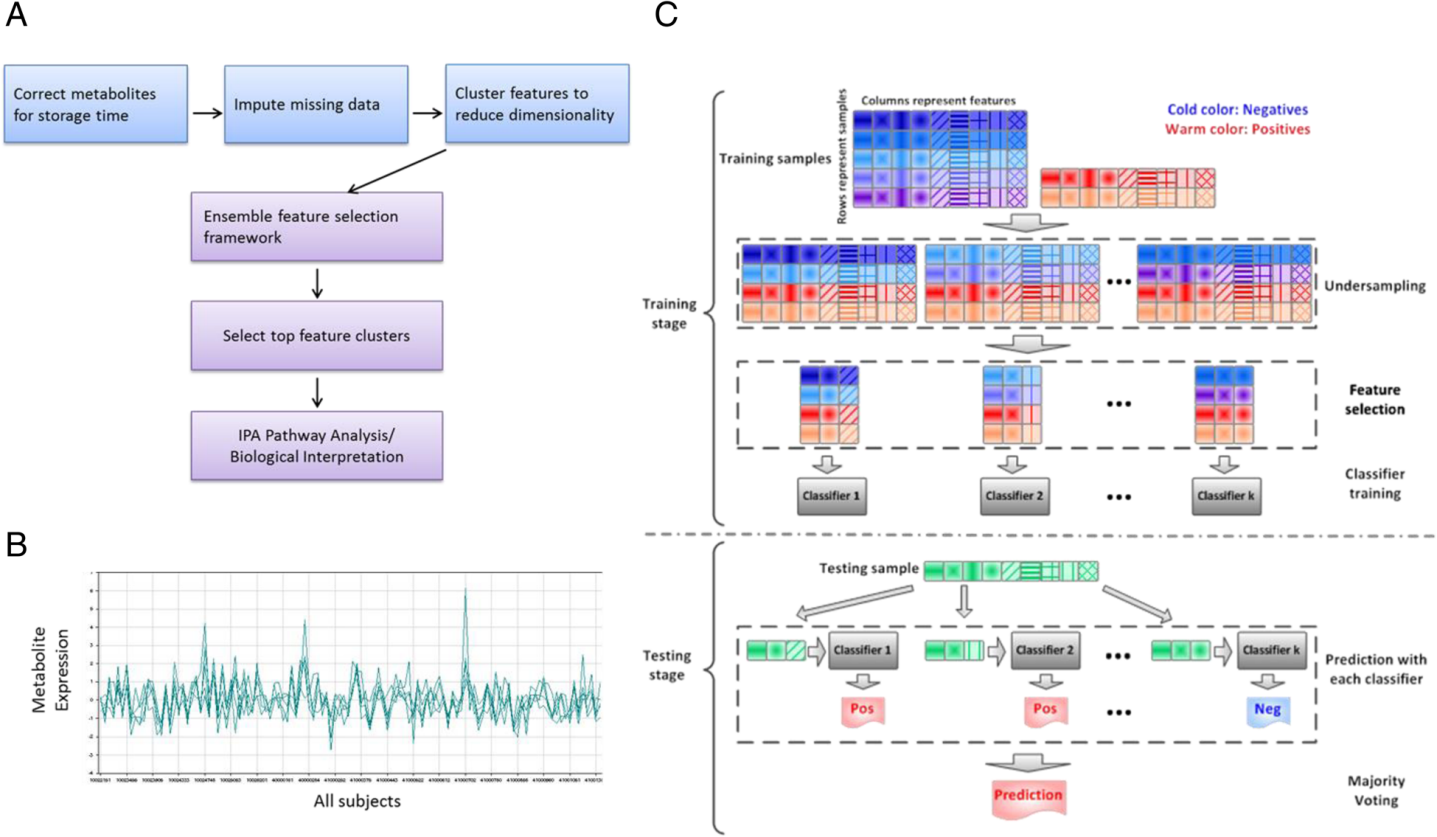
Analytic workflow aimed at maximizing both predictive power and biological interpretability. **a** Metabolite preprocessing includes (1) correction of each individual metabolite for storage time effects, (2) imputation of missing data, (3) feature clustering, (4) Classification using ensemble learning framework, (5) selection of top clusters/features, (6) pathway analysis and biological interpretation. **b** Example of cluster containing 5 metabolites. Highly correlated features are grouped into clusters using K-means or Heirarchical clustering. For each cluster, the cluster centroid is computed and used as a feature for ensebml learning. Subsequent pathway analysis includes all members of top clusters. **c** Illustration of the ensemble learning framework. Given the imbalanced training data, we randomly undersample the training data ***k*** times, and then we perform feature selection and classification on each undersampled dataset. Finally, we combine all ***k*** classifiers to make the final prediction, and report out top cluster-features

We next focused on optimizing the classification of the Melancholic Depressed subjects from Healthy Controls. Classifiers were built using either individual metabolites (228 features), or deriving features by first clustering metabolites using K-means or hierarchical clustering into 100 cluster. The 100 cluster centroids were used as features in the predictive models (Fig. 2b).

The Ensemble Feature Selection Framework (EFSF) was run testing four imputation methods (halfMin, kNN3, EM and SVD, described in the Methods), and four feature selection methods (Fisher’s Score, Gini Index, *T*-Test, and Stability Selection as described in the Methods). Tables 2 and 3 show the classification results by Random Forest and SVM, respectively, across all imputation, feature selection and classification methods. We observed that Random Forest achieved higher classification performance than SVM in most cases.

**Table 2:** Comparison of the classification performance obtained by Random Forest. For three clustering strategies, we compare 4 different imputation methods: halfMin, kNN3, EM, and SVD. And four feature selection methods: Fisher, Gini, *T*-test and Stability. These are described in the Methods. The method used for subsequent pathway analysis is in bold

Imputation	halfMin				kNN3				EM				SVD			
FS method	Fisher	Gini	*T*-test	Stability	Fisher	Gini	*T*-test	Stability	Fisher	Gini	*T*-test	Stability	Fisher	Gini	*T*-test	Stability
Raw Features																
Accuracy	80.42 %	80.36 %	80.42 %	80.34 %	78.68 %	80.43 %	77.84 %	83.84 %	77.84 %	76.87 %	77.84 %	78.74 %	77.84 %	76.11 %	77.84 %	81.18 %
Sensitivity	73.33 %	76.67 %	73.33 %	76.67 %	73.33 %	76.67 %	73.33 %	76.67 %	73.33 %	71.67 %	73.33 %	68.33 %	73.33 %	66.67 %	73.33 %	76.67 %
Specificity	82.22 %	81.22 %	82.22 %	81.11 %	80.22 %	81.33 %	79.11 %	85.44 %	79.11 %	78.00 %	79.11 %	81.11 %	79.11 %	78.11 %	79.11 %	82.11 %
Cluster-Representatives (K-means)																
Accuracy	77.92 %	80.50 %	77.92 %	79.51 %	**78.69 %**	**79.74 %**	**78.69 %**	**79.52 %**	77.07 %	77.16 %	75.34 %	74.15 %	78.74 %	78.74 %	80.48 %	79.73 %
Sensitivity	83.33 %	88.33 %	83.33 %	86.67 %	**76.67 %**	**81.67 %**	**81.67 %**	**81.67 %**	78.33 %	78.33 %	78.33 %	75.00 %	78.33 %	78.33 %	73.33 %	78.33 %
Specificity	77.22 %	79.22 %	77.22 %	78.11 %	**79.33 %**	**79.44 %**	**78.22 %**	**79.22 %**	77.11 %	77.22 %	75.11 %	74.33 %	79.11 %	79.11 %	82.33 %	80.33 %
Cluster-Representatives (Hierarchical Clustering)																
Accuracy	78.06 %	75.48 %	78.06 %	77.01 %	79.59 %	79.66 %	80.50 %	77.24 %	73.79 %	73.79 %	73.73 %	69.62 %	75.47 %	74.64 %	76.38 %	70.38 %
Sensitivity	73.33 %	78.33 %	73.33 %	83.33 %	78.33 %	83.33 %	78.33 %	78.33 %	73.33 %	80.00 %	73.33 %	70.00 %	70.00 %	70.00 %	70.00 %	70.00 %
Specificity	79.33 %	75.22 %	79.33 %	76.00 %	80.11 %	79.22 %	81.22 %	77.33 %	74.00 %	72.89 %	74.00 %	69.89 %	77.11 %	76.11 %	78.22 %	70.89 %

**Table 3:** Comparison of the classification performance obtained by Support Vector Machines. For three clustering strategies, we compare 4 different imputation methods: halfMin, kNN3, EM, and SVD. And four feature selection methods: Fisher, Gini, *T*-test and Stability. These are described in the Methods

Imputation	halfMin				kNN3				EM				SVD			
FS method	Fisher	Gini	*T*-test	Stability	Fisher	Gini	*T*-test	Stability	Fisher	Gini	*T*-test	Stability	Fisher	Gini	*T*-test	Stability
Raw Features																
Accuracy	72.13 %	79.52 %	72.13 %	74.64 %	71.08 %	77.77 %	72.74 %	75.68 %	71.22 %	78.68 %	74.56 %	73.73 %	71.99 %	78.74 %	71.85 %	75.54 %
Sensitivity	65.00 %	71.67 %	65.00 %	70.00 %	65.00 %	71.67 %	65.00 %	70.00 %	60.00 %	73.33 %	60.00 %	65.00 %	65.00 %	73.33 %	68.33 %	75.00 %
Specificity	74.11 %	81.33 %	74.11 %	76.11 %	72.78 %	79.00 %	74.78 %	77.22 %	74.00 %	80.11 %	78.11 %	76.00 %	73.89 %	80.11 %	72.78 %	76.11 %
Cluster-Representatives (K-means)																
Accuracy	77.83 %	80.20 %	78.68 %	79.73 %	78.06 %	80.57 %	79.66 %	76.46 %	72.96 %	71.14 %	73.79 %	74.00 %	79.65 %	79.43 %	79.65 %	72.67 %
Sensitivity	78.33 %	86.67 %	81.67 %	78.33 %	80.00 %	83.33 %	83.33 %	75.00 %	65.00 %	70.00 %	70.00 %	70.00 %	78.33 %	76.67 %	78.33 %	71.67 %
Specificity	78.00 %	79.00 %	78.11 %	80.33 %	78.22 %	80.44 %	79.22 %	77.33 %	75.00 %	71.78 %	75.00 %	75.11 %	80.11 %	80.00 %	80.11 %	72.78 %
Cluster-Representatives (Hierarchical Clustering)																
Accuracy	76.15 %	79.28 %	76.23 %	77.01 %	77.92 %	80.64 %	77.92 %	74.70 %	72.68 %	74.55 %	72.68 %	71.23 %	75.96 %	78.60 %	76.79 %	70.10 %
Sensitivity	85.00 %	83.33 %	85.00 %	78.33 %	78.33 %	75.00 %	78.33 %	75.00 %	80.00 %	80.00 %	80.00 %	75.00 %	63.33 %	70.00 %	73.33 %	68.33 %
Specificity	74.78 %	78.78 %	74.89 %	77.11 %	78.11 %	82.33 %	78.11 %	75.11 %	71.67 %	73.78 %	71.67 %	71.00 %	79.00 %	81.00 %	77.89 %	70.78 %

Imputing missing values with half of the minimum feature value achieved better performance than other methods on individual metabolite features, while K-nearest neighbor imputation outperforms others on cluster-representatives (both K-means and hierarchical clustering). The performance obtained by the EM imputation is slightly worse. However, overall all imputation methods achieve fairly comparable classification performance, a robustness that may be conferred by the small amount of missingness in the data set.

Stability selection, which penalizes highly correlated features that are less likely to be selected together in a model, performed consistently better on the individual features (not clustered). In contrast, Gini Index performed better when learning was performed on cluster-centroids.

Classification performance (especially sensitivity) overall was higher on K-means-clustered features than when using individual metabolite features; this indicates that highly correlated features with high predictive power can be effectively grouped by clustering approaches.

We also compare the EFSF framework with the standard undersampling method using exactly the same training-testing data splitting. The result of the standard undersampling method depends on which majority samples are selected. To get a good estimate of the performance of the standard undersampling method, we simply use the *k* classifiers from the *k* undersampled (balanced) data constructed in the EFSF and report the average performance of these *k* classifiers. The results are shown in Additional file 1: Tables S1 and S2 for Random Forest and Support Vector Machines, respectively. We observe that, the highest performance (accuracy, sensitivity, and specificity) achieved is around 73 %, which is significantly lower than that obtained by the EFSF.

**Fig. 3:**
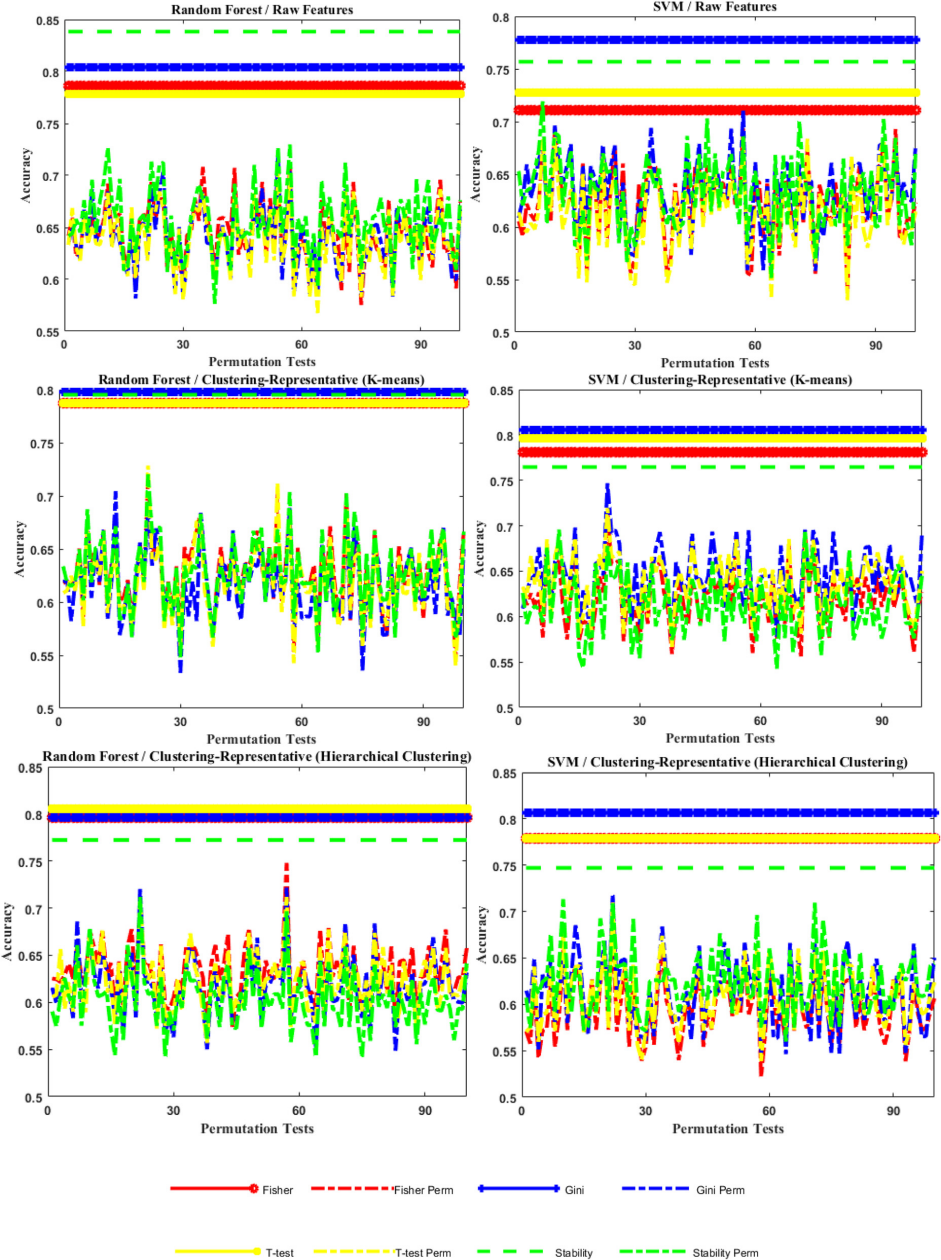
Classification accuracy obtained by Random Forest and SVM in permutation tests. The missing values in the data set are imputed via kNN3 method. The performance obtained in the original experiment is shown for reference

To further validate strength of the signals discovered in the study, we permutations tests (100 iterations, randomly permuting sample labels) and find that, for all cases, the classifier consistently performs better in the original data than in the permuted data and the permutation-based *p*-value is 1/101, which indicates that the performances achieved in the original study is significantly high with significant level α = 0.01 (Fig. 3). This suggests our classification performance is not the simple result of over-fitting to a small number of samples.

### The blood metabolomic biosignature of melancholic depression

We selected K-means clustering, kNN3 imputation with Random Forest classification as the combination method with highest overall classification performance on which to perform pathway analysis. Each feature selection method resulted in similar classification accuracy (range of 78.69–79.74 %), although cluster representatives selected by each method did not overlap completely. The number of cluster representatives required for optimal performance also varied; 15, 30, 36, and 18 for Gini, Stability, Fisher, and *t*-test, respectively. Features comprising the top 15 clusters ranked by score were selected from each method for evaluation of the biosignature. The union metabolites from the 4 methods was used, as each feature selection method resulted in similar classification accuracy and was, therefore, hypothesized to contribute biological information of interest. This yielded 76 metabolites, 48 of which were selected by at least 3 of the 4 methods. Stability Selection exhibited the least overlap with other methods, identifying 19 unique metabolites.

Fifty-six of the 76 metabolites were mapped to KEGG identifiers and analyzed. Table 3 lists the unique metabolites with their ontology class and the cluster ranking by each feature selection method. Top ranked features selected by other combinations of the ensemble framework parameters are included in Additional file 1: Tables S3-S7. A high degree of robustness among the top features is observed.

Many of the clusters were comprised of metabolites falling within the same ontology class (Table 4), suggesting that expression of classes of metabolites often co-vary with each other. As expected, performing classification with cluster representatives retained metabolites which could be biologically relevant, but which would have been excluded as redundant information using other classification methods.

**Table 4:** Unique metabolites included in pathway analysis. Metabolites from the kNN3 imputed, K-means clustering, were analyzed using IPA. Standardized KEGG nomenclature is included together with metabolite class and ranking of cluster representatives in the four feature selection methods. Metabolites were selected for pathway analysis if they were members of a cluster that was among the top 15 cluster-centroids selected by GINI, Stability, Fisher, or *T*-Test. Of 76 selected metabolites, 48 were selected by 3 of the 4 methods. 19 of the remainder were selected only by Stability

Metabolite Name	KEGG	Metabolite class	Cluster rank			
			Gini	Stability	Fisher	*T*-test
Triacylgyceride hydroperoxide (C18:1,18:2,C18:2-OOH)						
(additional: Triacylgyceride hydroperoxide C16:0,C18:1,C20:4-OOH,						
Triacylgyceride hydroperoxide (C18:1,C18:1,C18:3-OOH)	--	Lipid Hydroperoxides	1	1	6	2
Triacylgyceride hydroperoxide (C16:0,C18:1,C18:2-OOH)	--	Lipid Hydroperoxides	1	1	6	2
Cysteine	C00097	Amino acids	2	2	1	1
Cystine	C00491	Amino acids	2	2	1	1
Pseudouridine	C02067	Nucleobases (and related)	2	2	1	1
Unknown(28100470)	--	Unknown lipid	3	22	8	12
Conjugated linoleic acid (C18:trans[10]cis[12]2:	--	Fatty acids	3	22	8	12
Heptadecanoic acid (C17:0)	--	Fatty acids	3	22	8	12
Phenylalanine	C00079	Amino acids	4	19	2	3
Lysine	C00047	Amino acids	4	19	2	3
Methionine	C00073	Amino acids	4	19	2	3
Tyrosine	C00082	Amino acids	4	19	2	3
Alanine	C00041	Amino acids	4	19	2	3
Histamine	C00388	Catecholamines and other monoamines	5	12	12	9
Serotonine	C00780	Catecholamines and other monoamines	5	12	12	9
Fumarate	C00122	Energy metabolism and related	6	4	3	4
Normetanephrine	C05589	Catecholamines and other monoamines	6	4	3	4
Sphingomyelin (dl8:l/C16:0)	C00550	Sphingolipids	7	17	16	22
Unknown(58100162)		Unknown polar	7	17	16	22
DAG C18:1, C18:2) [seel]		Glycerides (Mono-, Di-, Triglycerides)	8	63	5	6
TAG (containing C16:l/C18:l or C16:0/C18:2;	C00042	Glycerides (Mono-, Di-, Triglycerides)	8	63	5	6
TAG (C55H10006) (e.g. C16:0,C18:1,C18:2)	C00042	Glycerides (Mono-, Di-, Triglycerides)	8	63	5	6
TAG (C55H9606) or (C50H10006) (e.g. C16:0,C18:2,C18:3 or C16:0,C16:0,C18:2	C00042	Glycerides (Mono-, Di-, Triglycerides)	8	63	5	6
TAG (containing C18:1/C18:2)	C00042	Glycerides (Mono-, Di-, Triglycerides)	8	63	5	6
TAG (C55H9806) (e.g. C16:0,C18:2,C18:2)	C00042	Glycerides (Mono-, Di-, Triglycerides)	8	63	5	6
Unknown(28100099)	--	Unknown lipid	9	11	4	14
Urea	C00086	Amino acids derivates	9	11	4	14
Unknown(68100044)	--	Unknown lipid	10	61	10	10
TAG (containing C18:2/C18:3)	C00042	Glycerides (Mono-, Di-, Triglycerides)	10	61	10	10
Unknown(68100059)	--	Unknown lipid	10	61	10	10
TAG (C55H10006) (e.g. C16:0,C18:1,C18:2) 1	C00042	Glycerides (Mono-, Di-, Triglycerides)	10	61	10	10
Linoleic acid (C18:cis[9,12]2	C01595	Fatty acids	10	61	10	10
TAG (containing C18:2,C18:2]	C00042	Glycerides (Mono-, Di-, Triglycerides)	10	61	10	10
Ceramide (dl8:l/C24:0)	C00195	Sphingolipids	11	83	9	7
Palmitic acid (C16:0)	C00249	Fatty acids	11	83	9	7
Oleic add (Cl8:cis[9] 1)	C00712	Fatty acids	11	83	9	7
Stearic acid (C18:0)	C01530	Fatty acids	11	83	9	7
Glycerol, lipid fraction	C00116	Cholesterol and fatty alcohols	11	83	9	7
Ceramide (dl8:l/C24:0)	C00195	Sphingolipids	11	83	9	7
Eicosenoic acid (C20:cis[ll]l)	C16526	Fatty acids	11	83	9	7
Dodecanol	C02277	Cholesterol and fatty alcohols	11	83	9	7
Metanephrfne	C05588	Catecholamines and other monoamines	12	7	11	8
Ribonic acid	C01685	Carbohydrates and related	13	45	17	18
myo-lnositol	C00137	Carbohydrates and related	13	45	17	18
Unknown(68100045)		Unknown lipid	14	57	13	13
Unknown(58100165)		Unknown polar	14	57	13	13
Phosphatidylcholine (C16:0/C18:2)	C00157	Phospholipids	14	57	13	13
Leucine	C00123	Amino acids	15	50	7	5
Valine	C00183	Amino acids	15	50	7	5
Isoleucine	C00407	Amino acids	15	50	7	5
TAG#1	C00042	Glycerides (Mono-, Di-, Triglycerides)	16	89	15	15
TAG (containing C16:0/C16:1 or C14:0/C18:l	C00042	Glycerides (Mono-, Di-, Triglycerides)	16	89	15	15
Palmitoleic acid	C08362	Fatty acids	16	89	15	15
Myristic acid (C14:0)	C06424	Fatty acids	16	89	15	15
Pentadecanol		Cholesterol and fatty alcohols	16	89	15	15
lndole-3-propionic acic		Amino acids derivates	17	13	22	16
Arginine	C00062	Amino acids	17	13	22	16
Elaidic acid	C01712	Fatty acids	18	10	14	11
Ratio Glu_versus_Gln	--	--	18	10	14	11
O-Phospho-L-tyrosine	C06501	Amino acids derivates	19	9	34	26
Unknown(58100024)	--	Unknown polar	19	9	34	26
Unknown(38100389)	--	Unknown polar	20	6	19	19
Unknown(38100468)	--	Unknown polar	20	6	19	19
Dopamine	C03758	Catecholamines and other monoamines	20	6	19	19
Unknown(68100052)	--	Unknown lipid	32	8	33	30
Arachidonic acid (C20:cis-[5,8,ll,14]4)	C00219	Fatty acids	32	8	33	30
Phosphatidylcholine #8	C00157	Phospholipids	32	8	33	30
Cholic acid	C00695	Miscellaneous	44	5	25	17
lndole-3-acetic acid	C00954	Amino acids derivates	44	5	25	17
Cortisol	COO735	Hormones and related	65	3	40	28
Corticosterone	C02140	Hormones and related	65	3	40	28
Androstendion	C00280	Hormones and related	65	3	40	28
Threonic acid	C01620	Vitamins, cofactors and related	74	15	57	58
Glyceric acid	C00258	Miscellaneous	74	15	57	58
Unknown(68100002)	--	Unknown lipid	76	14	42	35
Lysophosphatidylcholine (16:0)	C04230	Phospholipids	76	14	42	35

**Fig. 4:**
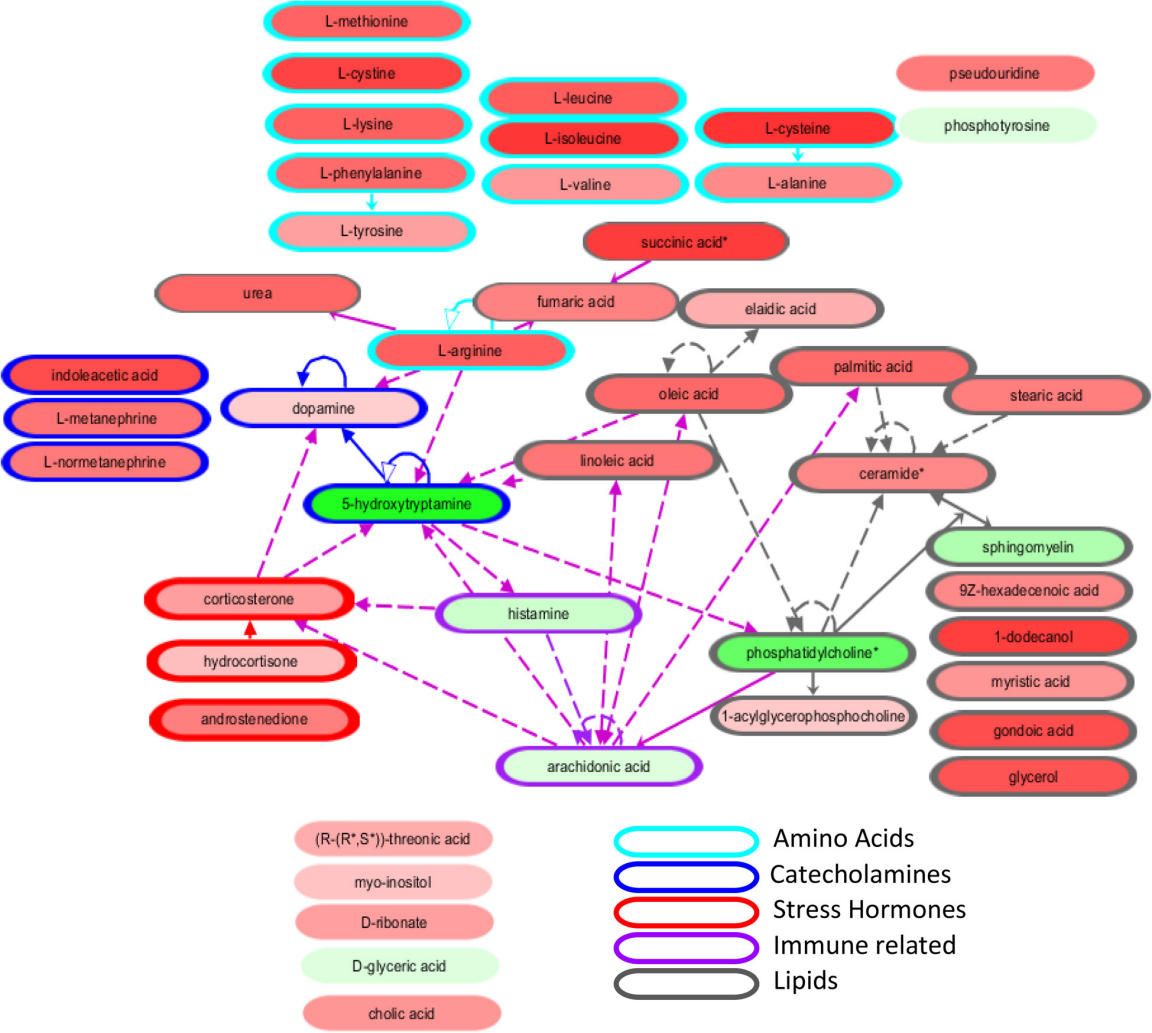
Network analysis of metabolites. IPA network analysis was used to view connections between selected metabolites. Several classes and biological functions were observed and are highlighted in the diagram. Red-filled metabolites are increased comparing melancholic depressed subjects to healthy controls, while green-filled metabolites are decreased. Higher intensity color indicated larger changes. Increases range from 1.94 to 1.07 fold change, while decreases range from -1.71 to -1.02

Overall, the majority of the selected metabolites were increased in melancholic depressed vs. healthy control subjects (48 of 56 metabolites). Some metabolite classes contribute greatly to the classification of melancholic depressed subjects from healthy controls (Fig. 4). Eleven of the 56 metabolites analyzed were amino acids (turquoise outlined metabolites), and several others (pseudouridine, phosphotyrosine, urea, fumaric acid, and succinic acid) are products of amino acid degradation or related. Fourteen of the metabolites are related to lipids (grey outlined metabolites) and most are increased in melancholic depressed subjects. While these changes suggest differences related to overall metabolism, there was no obvious mechanism revealed by canonical pathway analysis for these classes of metabolites.

Some of the metabolites suggested functional pathways of interest. Several are involved in catecholamine synthesis and degradation (outlined in blue), which have been studied for the treatment of depression. Three metabolites related to stress hormone signaling (outlined in red) were also identified as increased in melancholic depression vs. healthy controls. There are also 2 immune-related molecules (outlined in purple) that are decreased in melancholic depressed subjects.

## Discussion

Several studies have sought to understand symptomatic differences between MDD subtypes with inconsistent conclusions. A recent NESDA study [23] identified 3 main symptomatic groups which seemed to approximate melancholic, atypical, and undifferentiated/mixed subtypes. Another meta-analysis concluded that there is too much heterogeneity in studies and not enough detailed symptomatic information available to classify subtypes of MDD [43]. A third group found that response to antidepressants could not be predicted based on depression subtypes [5]. The variability of these studies suggests that symptomatic classification of MDD may not always reflect a shared etiology in depression subtypes. Several studies, however, point to factors such as severity of MDD, heritability, chronicity, and traumatic childhood events as being characteristic and predictive of melancholic depression [21, 23]. The fact that melancholia has a strong heritable component suggests that there is an underlying biological dysfunction which could be represented by molecular features and which could also be used to classify melancholic depressed subjects from other MDD subtypes or healthy controls. In our study, we have identified blood metabolite markers which are able to distinguish melancholic subjects from healthy controls with 80 % accuracy.

In our empirical study, the ensemble feature selection framework has shown great effectiveness in both feature selection and classification on imbalanced metabolite data. We thus expect it to be a promising framework capable of achieving robust classification results for the analysis of other biomedical data where the sample distribution is severely imbalanced. Based on the EFSF framework, we identified discriminative metabolite features related to melancholic depression, which are biologically relevant to the disease state.

We found that different methods of data imputation and feature selection had only minor effects on the accuracy of classification, suggesting that there are robust metabolite changes that can classify Melancholic Depressed subjects from Healthy Controls. Interestingly, although the overall accuracy of classification was similar, Stability feature selection identified several different metabolites than other methods. Different from other feature selection methods, stability selection identifies features which are stable under data perturbation (via subsampling/bootstrapping). Because all of the methods resulted in similar accuracy, we hypothesized that all of the metabolites were important contributors to melancholia and applied pathway analysis to understand biological functions that can contribute to the disease.

Despite characteristics of slowed motor movement, melancholic depression is described as a physiological state of hyperarousal, which includes a chronic activation of the hypothalamic–pituitary–adrenal axis. This stress pathway has been studied extensively in melancholia and in animal models of chronic stress (reviewed in [21, 44]). Elevated levels of the glucocorticoid, cortisol (also called hydrocortisone), is an important signaling molecule in this pathway and is increased in numerous studies of melancholia. Although cortisol changes are present in multiple subtypes of MDD, a recent meta-analysis of 354 studies found that the effect size for cortisol was larger when restricted to just melancholic depressed subjects [45]. Consistent with other studies, we identified an increase in cortisol in melancholia vs healthy controls. However, because we took a metabolomic approach, we also identified increases in other metabolites in the hormone biosynthesis pathway (androstenedione and corticosterone). Together these data might suggest a dysregulation promoting steroidogenesis in the upstream pathway. Glucocorticoids are also important anti-inflammatory molecules. There is renewed interest in the involvement of inflammation in the development of MDD, and it has been hypothesized that there is immune repression in melancholia mediated by chronic cortisol levels and activated inflammation in atypical depression [21, 46]. Our results are consistent with immune repression, evidenced by lower levels of histamine and arachidonic acid in melancholic depressed compared to healthy control subjects.

We also saw increases in catecholamine pathways, which are inherently linked to glucocorticoids through a complex feedback system. Lamers et al. (reviewed in [21]) have postulated that melancholic depression is a state of chronic stress, which activates corticotropin releasing hormone (CRH), cortisol, and norepinephrine (NE) pathways in the absence of inhibitory feedback. Several metabolites in this pathway were removed from our dataset due to instability over long storage times, including NE itself. However, NE’s precursor, dopamine, and metabolites of NE (L-metanephrine and L-normetanephrine) were increased in melancholic depressed subjects, which could suggest that NE may also be elevated. Another member of the catecholamine pathway is serotonin, which is decreased in our dataset. An important class of antidepressants, selective serotonin reuptake inhibitors (SSRI), is aimed at increasing extracellular levels of serotonin to treat depression. A decrease in plasma serotonin, as seen in these melancholic subjects, would be hypothesized to contribute to a depressive state.

Heart disease and type II diabetes (T2DM) have been cited as comorbidities of depression, which increases the mortality rate in depressed subjects independent of suicide. One shared mechanism for heart disease and T2DM is dyslipidemia characterized by increased triglycerides and fatty acids. Metabolic factors have been hypothesized to be associated with atypical depression, because of an increase in appetite and body mass index (BMI) [23]. The POWER study [4], however, found significant increases in triglycerides, ACTH, and leptin in a melancholic, but not atypical depression, cohort. Conversely, at least one study found no differences in triglycerides between melancholic and healthy subjects [46]. We found that both triglycerides and fatty acids were increased in melancholic depressed subjects compared to healthy controls, even though there was no difference in BMI between the groups (data not shown). Clusters containing triglycerides were consistently ranked highly by 3 of the 4 feature selection methods, indicating that they were important in classification of melancholia. Although not measured in this study, inflammatory factors such as cytokines may also play a role in development of insulin resistance in these subjects.

Two modified triglycerides (triglyerceride hydroperoxides) are particularly interesting as they were highly ranked in each of the feature selection methods. There is little literature about lipid hydroperoxides, although a recent study correlated an increase in lipid hydroperoxides and an inflammatory marker, c-reactive protein (CRP), in depressed smokers [47]. Lipid hydroperoxides are hypothesized to occur physiologically in plasma under conditions of oxidative stress and may also play a role in the development of heart disease [48]. In the NESDA dataset, the melancholic group contained a statistically significant increase in smokers over other types of depression [23]. We were unable to explore this correlation as our study did not record smoking status.

Amino acids are another class of metabolites that is consistently increased in this study of melancholic depression. While a mechanism for elevated amino acids cannot be determined from this dataset, there is evidence that this class of metabolites can differentiate MDD from healthy subjects. Branched chain amino acids were decreased following treatment with the selective serotonin reuptake inhibitor antidepressant sertraline [49]. Another recent study concludes that a combination of tryptophan, cysteine, and glutamine are capable of differentiating MDD from controls, although their results, unlike ours, identify a decrease in these plasma amino acids [50]. The inconsistency in directionality of amino acid changes potentially arises from the difference in study populations, where Xu et al. [50] examined MDD, and our results are determined from the melancholic subtype, which comprises only ~20 % of MDD patients in our study.

## Conclusions

This paper explores the metabolic biomarkers for melancholic depression, a major subtype of MDD. A comprehensive multivariate study on the identification of metabolic biomarkers that are strongly related to melancholic depression is of great importance and necessity for understanding the illness and the development of pharmaceuticals and therapies. To address the problems of confounding effects, incomplete data, feature correlation, and imbalanced sample distributions, we explored different data correction, imputation, and feature grouping methods with an ensemble feature selection framework for identifying biomarkers and building prediction model. Our extensive experiments on the metabolite data show that strong signals exist in metabolite data that can differentiate melancholic depressive patients from healthy controls. Our pathway analysis on differentiating metabolites elucidates underlying molecular mechanisms that could contribute to melancholic depression. We expect that the proposed computational system can be adapted to analyze other biomedical data with similar characteristics, which are common in many biomedical applications.

## Abbreviations

BMI, body mass index; BRC, Brain Resource Company; CRH, corticotropin releasing hormone; EFSF, ensemble feature selection framework; EM, expectation-maximization; halfMin, half of the minimum value; HC, healthy control; JRD, Janssen Research & Development, L.L.C; kNN, k nearest neighbor method; MDD, major depressive disorder; NE, norepinephrine; PMD, psychomotor disturbance; SVD, singular value decomposition; T2DM, type II diabetes

## Additional file

Additional file 1: Figure S1. Effect of storage time correction. **Figure S2.** The distributions of original and imputed metabolite features: Glyoxylate ratio, Caffeine ratio, Elaidicacid ratio and Indole 3 propionic acid ratio. **Figure S3.** QQ plots of the *p*-values of the two-sample t-tests on raw features, k-means and hierarchical clustering representatives. **Table S1.** Classification performance obtained by Random Forest on metabolite data using the standard undersampling technique. **Table S2.** Classification performance obtained by Support Vector Machines on metabolite data using the standard undersampling technique. **Table S3.** Top 30 individual metabolic features selected by different feature selection methods. **Table S4.** Top 30 individual metabolic features selected by different feature selection methods. **Table S5.** Top cluster-representatives (K-means) selected by different feature selection methods. **Table S6.** Top cluster-representatives (hierarchical clustering) selected by different feature selection methods. **Table S7.** Top cluster-representatives (hierarchical clustering) selected by different feature selection methods. (DOCX 812 kb)

## References

[1] WHO. DEPRESSION: A Global Crisis. World Federation for Mental Health. http://www.who.int/mental_health/management/depression/wfmh_paper_depression_wmhd_2012.pdf.

[2] Fava M, Rush AJ, Trivedi MH, Nierenberg AA, Thase ME, Sackeim HA, Quitkin FM, Wisniewski S, Lavori PW, Rosenbaum JF (2003). Background and rationale for the sequenced treatment alternatives to relieve depression (STAR^* D) study. Psychiatr Clin N Am.

[3] Ripke S, Wray NR, Lewis CM, Hamilton SP, Weissman MM, Breen G, Byrne EM, Blackwood DH, Boomsma DI, Cichon S (2013). A mega-analysis of genome-wide association studies for major depressive disorder. Mol Psychiatry.

[4] Cizza G, Ronsaville DS, Kleitz H, Eskandari F, Mistry S, Torvik S, Sonbolian N, Reynolds JC, Blackman MR, Gold PW (2012). Clinical subtypes of depression are associated with specific metabolic parameters and circadian endocrine profiles in women: the power study. PLoS One.

[5] Uher R, Dernovsek MZ, Mors O, Hauser J, Souery D, Zobel A, Maier W, Henigsberg N, Kalember P, Rietschel M (2011). Melancholic, atypical and anxious depression subtypes and outcome of treatment with escitalopram and nortriptyline. J Affect Disord.

[6] Rush A, Trivedi M, Wisniewski S, Nierenberg A, Stewart J, Warden D, Niederehe G, Thase M, Lavori P, Lebowitz B (2006). Acute and longer-term outcomes in depressed outpatients requiring one or several treatment steps: a STAR* D report. Am J Psychiatry.

[7] van Praag HM (2008). Kraepelin, biological psychiatry, and beyond. Eur Arch Psychiatry Clin Neurosci.

[8] Klein DN (2008). Classification of depressive disorders in DSM-V: Proposal for a two-dimension system. J Abnorm Psychol.

[9] Joyce PR (2008). Classification of mood disorders in DSM-V and DSM-VI. Australas Psychiatry.

[10] Halbreich U (2006). Major depression is not a diagnosis, it is a departure point to differential diagnosis—clinical and hormonal considerations:(A commentary and elaboration on Antonejevic’s paper). Psychoneuroendocrinology.

[11] Antonijevic IA (2006). Depressive disorders—is it time to endorse different pathophysiologies?. Psychoneuroendocrinology.

[12] Parker G, Fink M, Shorter E, Taylor MA, Akiskal H, Berrios G, Bolwig T, Brown WA, Carroll B, Healy D (2010). Issues for DSM-5: whither melancholia? The case for its classification as a distinct mood disorder. Am J Psychiatry.

[13] Rush AJ, Weissenburger JE (1994). Melancholic symptom features and DSM-IV. Am J Psychiatry.

[14] Gili M, Roca M, Armengol S, Asensio D, Garcia-Campayo J, Parker G (2012). Clinical patterns and treatment outcome in patients with melancholic, atypical and non-melancholic depressions. PloS one.

[15] Parker G, Hadzi-Pavlovic D, Boyce P. Issues in classification: II. Classifying melancholia. Melancholia. 1996:20-37.

[16] Parker G, McCraw S, Blanch B, Hadzi-Pavlovic D, Synnott H, Rees A-M (2012). Discriminating melancholic and non-melancholic depression by prototypic clinical features. J Affect Disord.

[17] Brown C, Battista DR, Sereika SM, Bruehlman RD, Dunbar-Jacob J, Thase ME (2007). Primary care patients’ personal illness models for depression: relationship to coping behavior and functional disability. Gen Hosp Psychiatry.

[18] Fava M (2008). A John Rush M, Alpert JE, Balasubramani G, Wisniewski SR, Carmin CN, Biggs MM, Zisook S, Leuchter A, Howland R. Difference in treatment outcome in outpatients with anxious versus nonanxious depression: a STAR* D report. Am J Psychiatry.

[19] Fawcett J (1997). The detection and consequences of anxiety in clinical depression. J Clin Psychiatry.

[20] Gaspersz R, Lamers F, Kent JM, Beekman A, Smit JH, van Hemert AM, Schoevers RA, Penninx B. Longitudinal predictive validity of the DSM-5 anxious distress specifier for clinical outcomes in a large cohort of patients with major depressive disorder. J Clin Psychiatry. 2016. 10.4088/JCP.15m10221 27035515

[21] Gold P, Chrousos G (2002). Organization of the stress system and its dysregulation in melancholic and atypical depression: high vs low CRH/NE states. Mol Psychiatry.

[22] Hickie I, Wilhelm K, Parker G, Boyce P, Hadzi-Pavlovic D, Brodaty H, Mitchell P (1990). Perceived dysfunctional intimate relationships: A specific association with the non-melancholic depressive subtype. J Affect Disord.

[23] Lamers F, de Jonge P, Nolen WA, Smit JH, Zitman FG, Beekman AT, Penninx BW (2010). Identifying depressive subtypes in a large cohort study: results from the Netherlands Study of Depression and Anxiety (NESDA). J Clin Psychiatry.

[24] Wong M-L, Kling MA, Munson PJ, Listwak S, Licinio J, Prolo P, Karp B, McCutcheon IE, Geracioti TD, DeBellis MD (2000). Pronounced and sustained central hypernoradrenergic function in major depression with melancholic features: relation to hypercortisolism and corticotropin-releasing hormone. Proc Natl Acad Sci.

[25] Monzón S, Gili M, Vives M, Serrano MJ, Bauza N, Molina R, García-Toro M, Salvà J, Llobera J, Roca M (2010). Melancholic versus non-melancholic depression: differences on cognitive function. A longitudinal study protocol. BMC Psychiatry.

[26] Parker G, Hadzi-Pavlovic D, Wilhelm K, Hickie I, Brodaty H, Boyce P, Mitchell P, Eyers K (1994). Defining melancholia: properties of a refined sign-based measure. Br J Psychiatry.

[27] Friston KJ, Holmes AP, Worsley KJ, Poline JP, Frith CD, Frackowiak RS (1994). Statistical parametric maps in functional imaging: a general linear approach. Hum Brain Mapp.

[28] Dukart J, Schroeter ML, Mueller K (2011). Age correction in dementia–matching to a healthy brain. PLoS One.

[29] Schneider T (2001). Analysis of incomplete climate data: Estimation of mean values and covariance matrices and imputation of missing values. J Climate.

[30] Bühlmann P, Rütimann P, van de Geer S, Zhang C-H (2013). Correlated variables in regression: clustering and sparse estimation. J Stat Plann Inference.

[31] Hastie T, Tibshirani R, Friedman J, Franklin J (2005). The elements of statistical learning: data mining, inference and prediction. Math Intell.

[32] He H, Garcia EA (2009). Learning from imbalanced data. Knowl Data Eng IEEE Trans.

[33] Liu X-Y, Wu J, Zhou Z-H (2009). Exploratory undersampling for class-imbalance learning. IEEE Trans Syst Man Cybern B Cybern.

[34] Dubey R, Zhou J, Wang Y, Thompson PM, Ye J (2013). Analysis of sampling techniques for imbalanced data: an n = 648 ADNI study. NeuroImage..

[35] Fan J, Fan Y (2008). High dimensional classification using features annealed independence rules. Ann Stat.

[36] Duda RO, Hart PE, Stork DG. Pattern classification. New York: John Wiley & Sons; 2000. p. 680. ISBN:978-0-471-05669-0.

[37] Gini C (1912). Italian: Variabilità e Mutabilità (Variability and Mutability).

[38] Meinshausen N, Bühlmann P (2010). Stability selection. J R Stat Soc B (Stat Methodol).

[39] Boser BE, Guyon IM, Vapnik VN. A Training Algorithm for Optimal Margin Classifiers. In: Proceedings of the Fifth Annual Workshop on Computational Learning Theory. COLT '92. New York: ACM; 1992. p. 144-52. Numpages 9. ISBN:0-89791-497-X. doi:10.1145/130385.130401.

[40] Breiman L (2001). Random forests. Mach Learn.

[41] Ojala M, Garriga GC (2010). Permutation tests for studying classifier performance. J Mach Learn Res.

[42] Good P. Permutation tests: a practical guide to resampling methods for testing hypotheses. Springer Science & Business Media. New York: Springer-Verlag; 1994. p. 228. ISBN:978-1-4757-2346-5.

[43] van Loo HM, de Jonge P, Romeijn J-W, Kessler RC, Schoevers RA (2012). Data-driven subtypes of major depressive disorder: a systematic review. BMC Med.

[44] Gold PW, Gabry KE, Yasuda MR, Chrousos GP (2002). Divergent endocrine abnormalities in melancholic and atypical depression: clinical and pathophysiologic implications. Endocrinol Metab Clin North Am.

[45] Stetler C, Miller GE (2011). Depression and hypothalamic-pituitary-adrenal activation: a quantitative summary of four decades of research. Psychosom Med.

[46] Lamers F, Vogelzangs N, Merikangas K, de Jonge P, Beekman A, Penninx B (2012). Evidence for a differential role of HPA-axis function, inflammation and metabolic syndrome in melancholic versus atypical depression. Mol Psychiatry..

[47] Vargas HO, Nunes SOV, Castro MRPd, Vargas MM, Barbosa DS, Bortolasci CC, Venugopal K, Dodd S, Berk M. Oxidative stress and inflammatory markers are associated with depression and nicotine dependence. Neurosci Lett. 2013;544:196–140. 10.1016/j.neulet.2013.03.059 23583694

[48] Frei B, Stocker R, Ames BN (1988). Antioxidant defenses and lipid peroxidation in human blood plasma. Proc Natl Acad Sci.

[49] Kaddurah-Daouk R, Bogdanov M, Wikoff W, Zhu H, Boyle S, Churchill E, Wang Z, Rush A, Krishnan R, Pickering E (2013). Pharmacometabolomic mapping of early biochemical changes induced by sertraline and placebo. Transl Psychiatry.

[50] Xu H-B, Fang L, Hu Z-C, Chen Y-C, Chen J-J, Li F-F, Lu J, Mu J, Xie P (2012). Potential clinical utility of plasma amino acid profiling in the detection of major depressive disorder. Psychiatry Res.

