# Supplementary material for: Metabolomic biosignature differentiates melancholic depressive patients from healthy controls
Source: BMC Genomics. 2016 Aug 23;17(1):669. doi: 10.1186/s12864-016-2953-2 (PMC4994306; doi:10.1186/s12864-016-2953-2)
Supplement: Additional file 1: Figure S1. — Effect of storage time correction. Figure S2. The distributions of original and imputed metabolite features: Glyoxylate ratio, Caffeine ratio, Elaidicacid ratio and Indole 3 propionic acid ratio. Figure S3. QQ plots of the p-values of the two-sample t-tests on raw features, k-means and hierarchical clustering representatives. Table S1. Classification performance obtained by Random Forest on metabolite data using the standard undersampling technique. Table S2. Classification performance obtained by Support Vector Machines on metabolite data using the standard undersampling technique. Table S3. Top 30 individual metabolic features selected by different feature selection methods. Table S4. Top 30 individual metabolic features selected by different feature selection methods. Table S5. Top cluster-representatives (K-means) selected by different feature selection methods. Table S6. Top cluster-representatives (hierarchical clustering) selected by different feature selection methods. Table S7. Top cluster-representatives (hierarchical clustering) selected by different feature selection methods. (DOCX 812 kb) [file 12864_2016_2953_MOESM1_ESM.docx]

**Supplementary Material**

**Supplmental Figures**


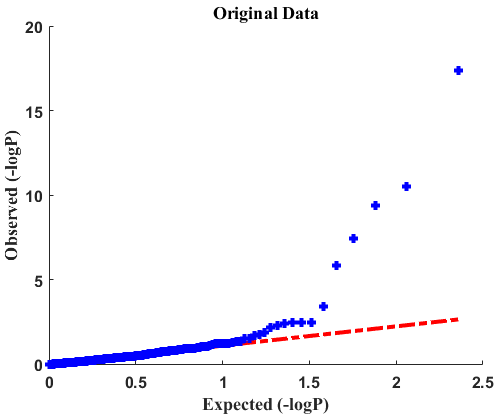

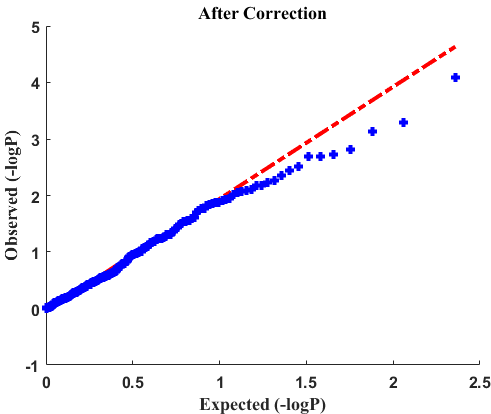


Additional file 1: Figure S1: Effect of storage time correction. Left: QQ plot of p-values of bivariate correlation between original metabolite features and storage time; Right: QQ plot of p-values of bivariate correlation between metabolite features and storage time after correcting effects of storage time based on HCs.


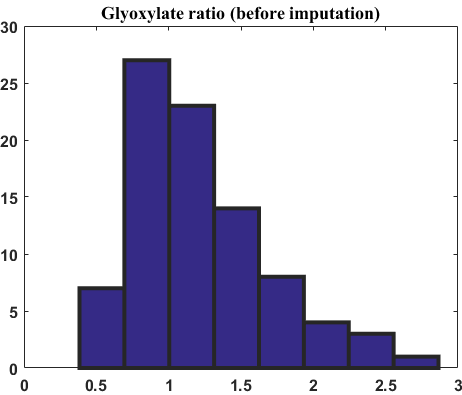

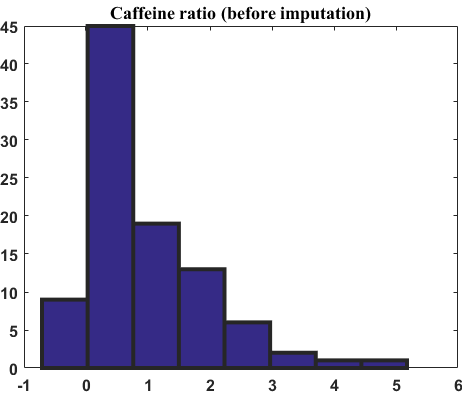

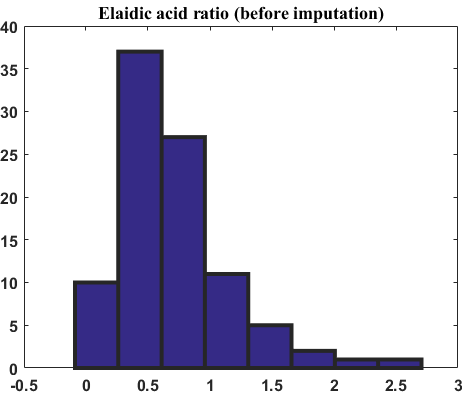

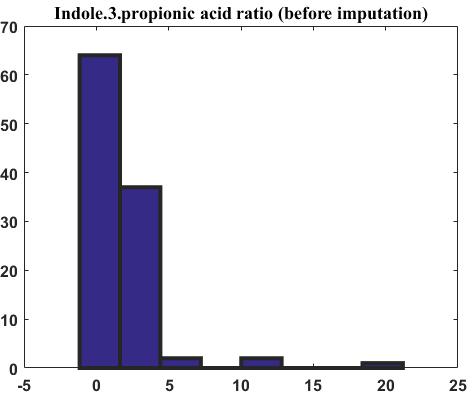


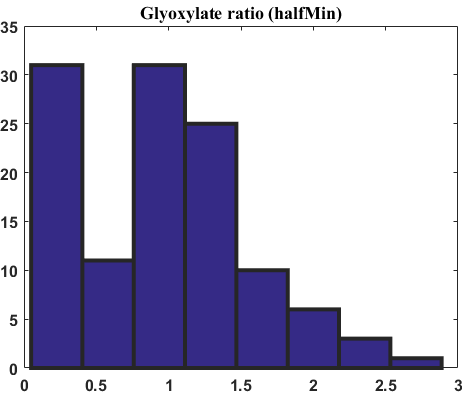

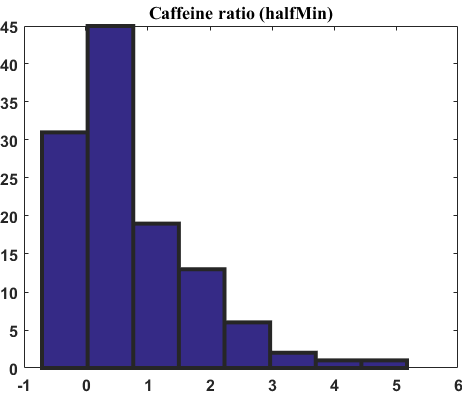

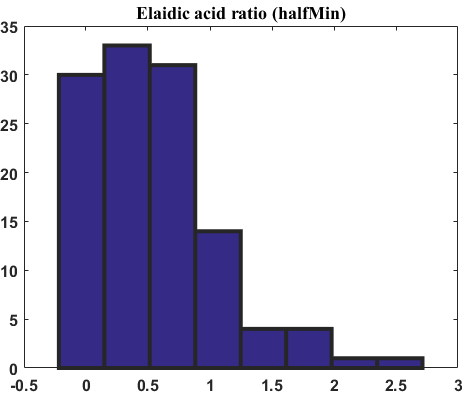

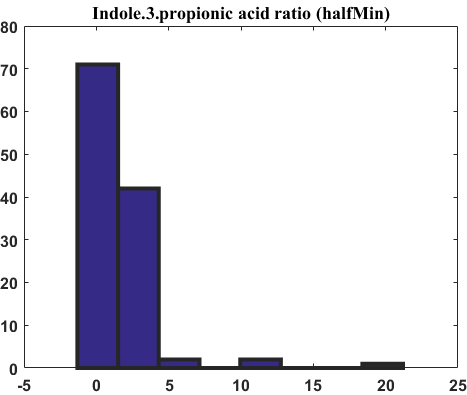


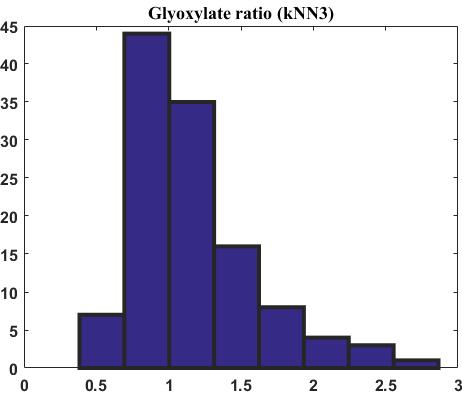

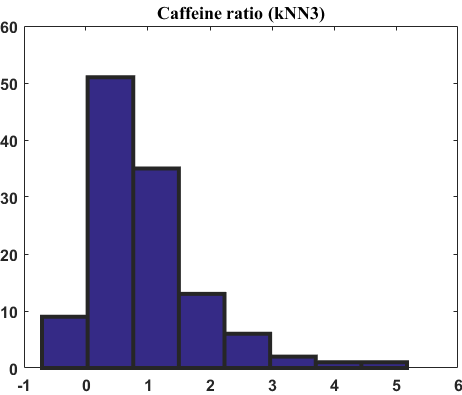

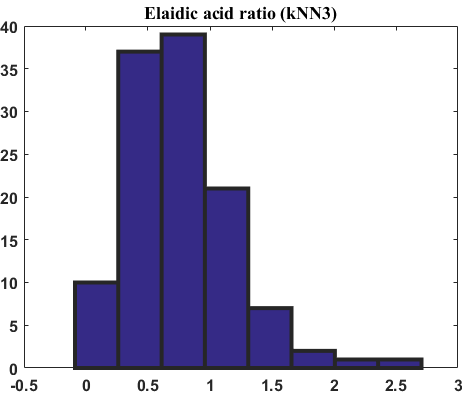

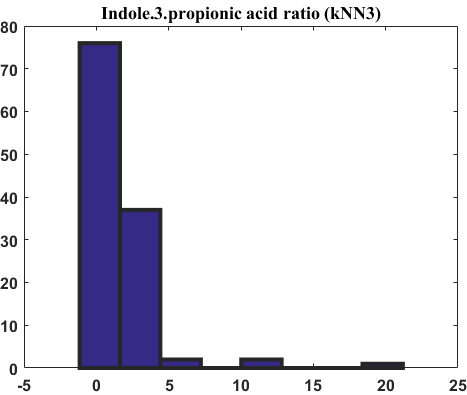


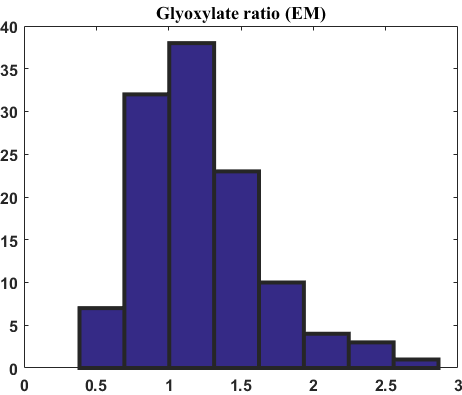

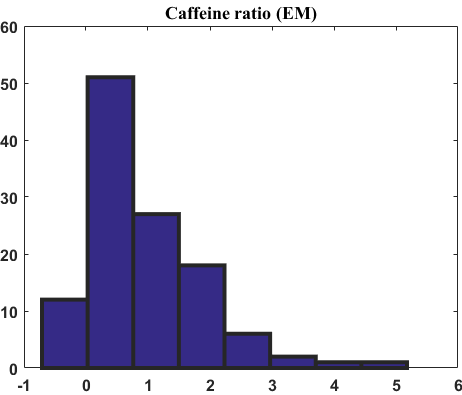

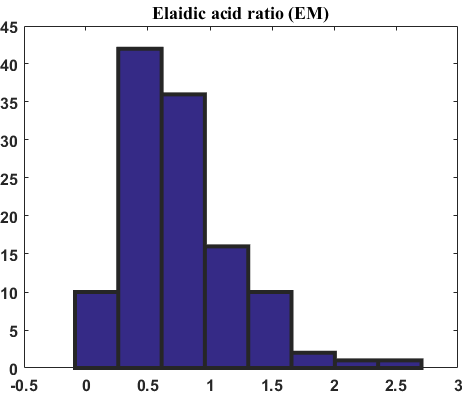

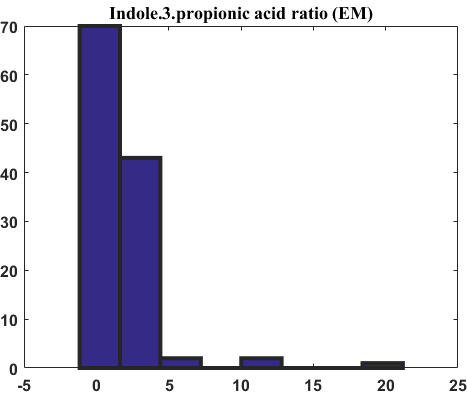


**
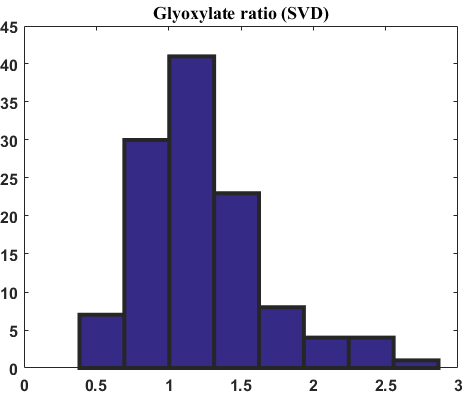

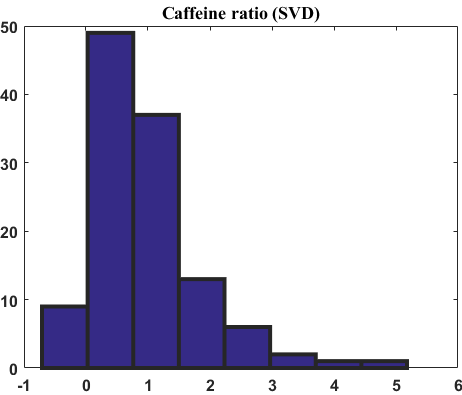

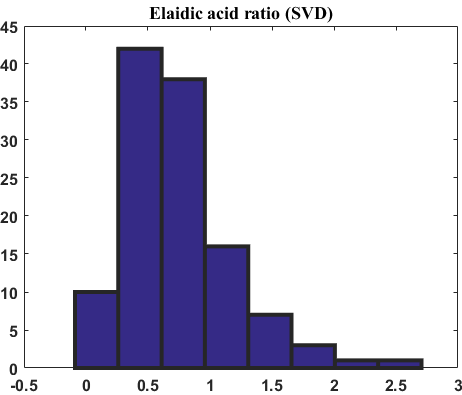

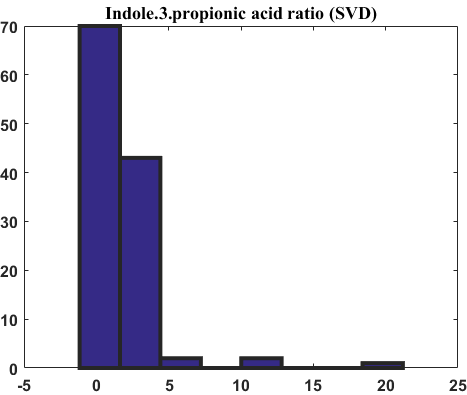
**

Additional file 1: Figure S2: The distributions of original and imputed metabolite features: Glyoxylate ratio, Caffeine ratio, Elaidicacid ratio and Indole 3 propionic acid ratio. The first row corresponds to the distribution of original feature values; the second row corresponds to the distribution of feature values imputed by the halfMin methods; the third row corresponds to the distribution of feature values imputed by the kNN3; the fourth row corresponds to the distribution of feature values imputed by the EM; the fifth row corresponds to the distribution of feature values imputed by the SVD.


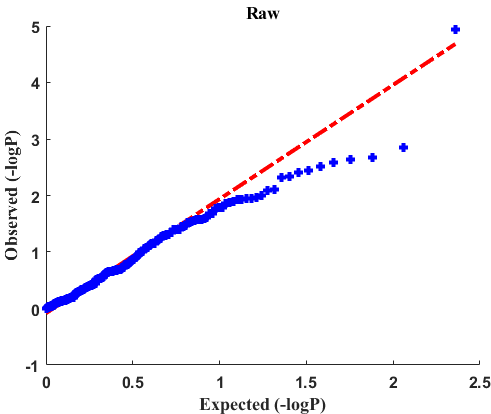

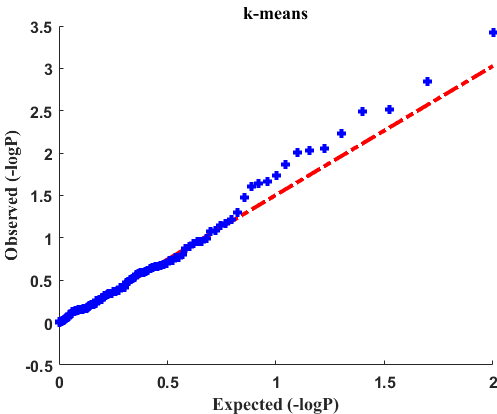

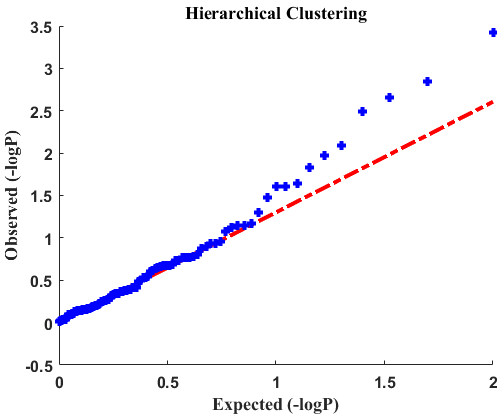


Additional file 1: **Figure S3. QQ plots of the p-values of the two-sample t-tests on raw features, k-means and hierarchical clustering representatives.** The missing values in the data set are imputed via kNN3 method.

Supplemental Tables

Additional file 1: Table S1. Classification performance obtained by Random Forest on metabolite data using the standard undersampling technique. We compare 2 imputation methods, half of minimum values (halfMin) and K-nearest-neighborhood (K=3, kNN3). We compare 4 feature selection methods including T-test, Fisher Information (Fisher), Gini, and stability selection (Stability).

| Raw Features | | | | | | | | | | | | | | | | |
| --- | --- | --- | --- | --- | --- | --- | --- | --- | --- | --- | --- | --- | --- | --- | --- | --- |
| Imputation | halfMin | | | | kNN3 | | | | EM | | | | SVD | | | |
| FS method | Fisher | Gini | T-test | Stability | Fisher | Gini | T-test | Stability | Fisher | Gini | T-test | Stability | Fisher | Gini | T-test | Stability |
| Accuracy | 73.60% | 72.76% | 73.60% | 71.39% | 74.54% | 73.73% | 74.54% | 73.04% | 73.30% | 71.88% | 73.30% | 71.82% | 73.26% | 71.81% | 73.26% | 71.71% |
| Sensitivity | 67.22% | 66.89% | 67.22% | 66.06% | 66.67% | 65.28% | 66.67% | 66.17% | 63.78% | 63.67% | 63.78% | 64.67% | 63.61% | 62.56% | 63.61% | 64.22% |
| Specificity | 75.14% | 74.15% | 75.14% | 72.67% | 76.36% | 75.66% | 76.36% | 74.66% | 75.44% | 73.73% | 75.44% | 73.46% | 75.41% | 73.87% | 75.41% | 73.40% |
| Cluster-Representatives (K-means) | | | | | | | | | | | | | | | | |
| Imputation | halfMin | | | | kNN3 | | | | EM | | | | SVD | | | |
| FS method | Fisher | Gini | T-test | Stability | Fisher | Gini | T-test | Stability | Fisher | Gini | T-test | Stability | Fisher | Gini | T-test | Stability |
| Accuracy | 73.44% | 73.28% | 73.44% | 71.35% | 73.52% | 72.71% | 73.52% | 72.09% | 70.72% | 70.64% | 70.72% | 68.25% | 73.32% | 72.81% | 73.32% | 72.07% |
| Sensitivity | 68.33% | 70.61% | 68.33% | 68.00% | 72.67% | 69.67% | 72.67% | 70.00% | 64.11% | 64.56% | 64.11% | 64.94% | 67.61% | 66.94% | 67.61% | 66.06% |
| Specificity | 74.73% | 74.07% | 74.73% | 72.20% | 73.88% | 73.57% | 73.88% | 72.72% | 72.34% | 72.16% | 72.34% | 69.13% | 74.73% | 74.30% | 74.73% | 73.56% |
| Cluster-Representatives (Hierarchical Clustering) | | | | | | | | | | | | | | | | |
| Imputation | halfMin | | | | kNN3 | | | | EM | | | | SVD | | | |
| FS method | Fisher | Gini | T-test | Stability | Fisher | Gini | T-test | Stability | Fisher | Gini | T-test | Stability | Fisher | Gini | T-test | Stability |
| Accuracy | 68.64% | 67.65% | 68.64% | 67.49% | 72.16% | 70.88% | 72.16% | 70.87% | 67.21% | 66.60% | 67.21% | 64.71% | 69.66% | 69.17% | 69.66% | 67.57% |
| Sensitivity | 66.89% | 63.00% | 66.89% | 65.50% | 71.22% | 67.72% | 71.22% | 69.50% | 63.06% | 63.89% | 63.06% | 61.11% | 66.06% | 65.89% | 66.06% | 65.11% |
| Specificity | 69.27% | 68.87% | 69.27% | 68.10% | 72.55% | 71.79% | 72.55% | 71.41% | 68.34% | 67.47% | 68.34% | 65.76% | 70.69% | 70.13% | 70.69% | 68.36% |

Additional file 1: Table S2. Classification performance obtained by Support Vector Machines on metabolite data using the standard undersampling technique. We compare 2 imputation methods, half of minimum values (halfMin) and K-nearest-neighborhood (K=3, kNN3). We compare 4 feature selection methods including T-test, Fisher Information (Fisher), Gini, and stability selection (Stability).

| Raw Features | | | | | | | | | | | | | | | | |
| --- | --- | --- | --- | --- | --- | --- | --- | --- | --- | --- | --- | --- | --- | --- | --- | --- |
| Imputation | halfMin | | | | kNN3 | | | | EM | | | | SVD | | | |
| FS method | Fisher | Gini | T-test | Stability | Fisher | Gini | T-test | Stability | Fisher | Gini | T-test | Stability | Fisher | Gini | T-test | Stability |
| Accuracy | 64.27% | 67.32% | 63.62% | 65.70% | 64.26% | 69.09% | 64.53% | 67.44% | 65.38% | 67.73% | 65.38% | 67.44% | 66.02% | 68.06% | 61.11% | 65.63% |
| Sensitivity | 57.22% | 62.17% | 58.39% | 57.11% | 57.06% | 63.17% | 56.06% | 57.78% | 57.56% | 60.22% | 57.56% | 58.28% | 58.83% | 61.67% | 51.44% | 59.50% |
| Specificity | 66.01% | 68.60% | 65.02% | 67.81% | 66.00% | 70.52% | 66.53% | 69.77% | 67.27% | 69.49% | 67.27% | 69.64% | 67.74% | 69.60% | 63.27% | 67.14% |
| Cluster-Representatives (K-means) | | | | | | | | | | | | | | | | |
| Imputation | halfMin | | | | kNN3 | | | | EM | | | | SVD | | | |
| FS method | Fisher | Gini | T-test | Stability | Fisher | Gini | T-test | Stability | Fisher | Gini | T-test | Stability | Fisher | Gini | T-test | Stability |
| Accuracy | 69.27% | 69.39% | 69.27% | 70.15% | 68.64% | 71.37% | 68.64% | 68.43% | 66.37% | 66.53% | 66.37% | 66.19% | 69.72% | 69.88% | 66.54% | 67.10% |
| Sensitivity | 61.94% | 69.94% | 61.94% | 62.11% | 63.61% | 68.28% | 63.61% | 60.61% | 61.28% | 62.94% | 61.28% | 65.44% | 68.00% | 64.28% | 61.72% | 68.28% |
| Specificity | 70.98% | 69.50% | 70.98% | 72.07% | 69.93% | 72.28% | 69.93% | 70.31% | 67.71% | 67.59% | 67.71% | 66.44% | 70.30% | 71.30% | 67.91% | 67.09% |
| Cluster-Representatives (Hierarchical Clustering) | | | | | | | | | | | | | | | | |
| Imputation | halfMin | | | | kNN3 | | | | EM | | | | SVD | | | |
| FS method | Fisher | Gini | T-test | Stability | Fisher | Gini | T-test | Stability | Fisher | Gini | T-test | Stability | Fisher | Gini | T-test | Stability |
| Accuracy | 67.80% | 67.98% | 67.80% | 65.79% | 68.31% | 72.47% | 68.31% | 66.51% | 66.36% | 65.18% | 66.36% | 64.84% | 69.07% | 68.28% | 69.07% | 65.96% |
| Sensitivity | 66.67% | 63.61% | 66.67% | 62.17% | 66.33% | 66.61% | 66.33% | 60.33% | 64.78% | 60.89% | 64.78% | 61.00% | 65.17% | 66.50% | 65.17% | 62.56% |
| Specificity | 68.29% | 69.17% | 68.29% | 66.72% | 68.91% | 73.98% | 68.91% | 68.02% | 66.94% | 66.33% | 66.94% | 65.87% | 70.13% | 68.94% | 70.13% | 66.96% |

Additional file 1: Table S3. Top 30 individual metabolic features selected by different feature selection methods. The data is imputed by kNN3. The ensemble learning takes place on individual metabolites.

Additional file 1: **Table S4. Top 30 individual metabolic features selected by different feature selection methods.** The data is imputed by halfMin. The ensemble learning takes place on individual metabolites.

Additional file 1: Table S5. Top cluster-representatives (K-means) selected by different feature selection methods. The data is imputed by halfMin.

Additional file 1: Table S6. Top cluster-representatives (hierarchical clustering) selected by different feature selection methods. The data is imputed by kNN3.

Additional file 1: Table S7. Top cluster-representatives (hierarchical clustering) selected by different feature selection methods. The data is imputed by halfMin.
